# Supplementary material for: Spin-valley coupling in single-electron bilayer graphene quantum dots
Source: Nat Commun. 2021 Sep 2;12:5250. doi: 10.1038/s41467-021-25498-3 (PMC8413270; doi:10.1038/s41467-021-25498-3)
Supplement: Supplementary file 1 — Supplementary Information [file 41467_2021_25498_MOESM1_ESM.pdf]

Supplementaray information: Spin-valley coupling in single-electron bilayer graphene quantum dots

L. Banszerus,[1,2,*] S. Möller,[1,2,*] C. Steiner,[1,2] E. Icking,[1,2] S. Trellenkamp,[3] F. Lentz,[3] K. Watanabe,[4] T. Taniguchi,[5] C. Volk,[1,2] and C. Stampfer[1,2,†]

[1]*JARA-FIT and 2nd Institute of Physics, RWTH Aachen University, 52074 Aachen, Germany, EU*

[2]*Peter Grünberg Institute (PGI-9), Forschungszentrum Jülich, 52425 Jülich, Germany, EU*

[3]*Helmholtz Nano Facility, Forschungszentrum Jülich, 52425 Jülich, Germany, EU*

[4]*Research Center for Functional Materials, National Institute for Materials Science, 1-1 Namiki, Tsukuba 305-0044, Japan*

[5]*International Center for Materials Nanoarchitectonics, National Institute for Materials Science, 1-1 Namiki, Tsukuba 305-0044, Japan*

(Dated: August 11, 2021)

[*] These two authors contributed equally.

[†] stampfer@physik.rwth-aachen.de

SUPPLEMENTARY NOTE 1: DETAILS ON SAMPLE DESIGN AND TUNING TOWARDS A SINGLE ELECTRON DQD

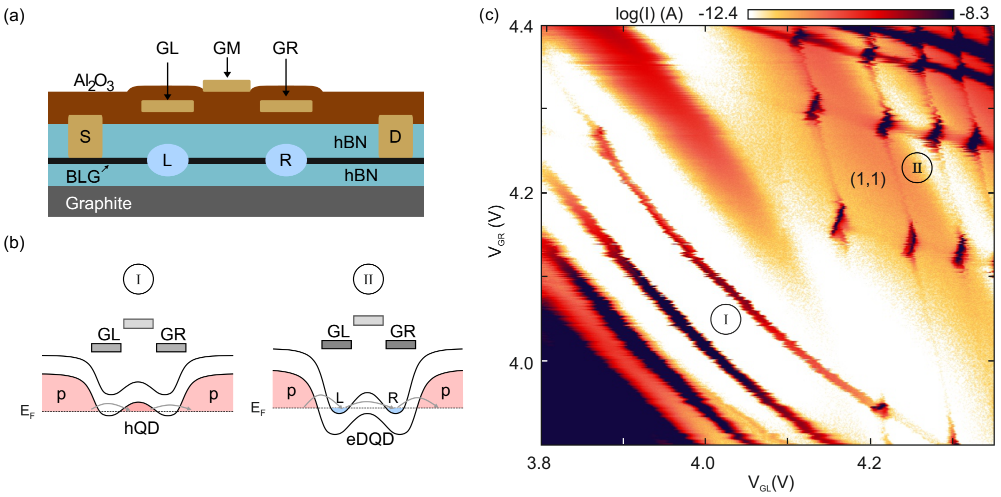

Supplementary Figure 1. **Device layout and tuning (a)** Schematic cross-section through the device. BLG is encapsulated between two flakes of hBN and placed on a graphite gate. Two Cr/Au contacts (S and D) are evaporated onto the BLG. Two layers of Cr/Au FGs are used to form a DQD. Atomic layer deposited Al_2O_3 serves as a gate dielectric. **(b)** Band alignment of the single QD and DQD regimes. **(c)** Charge stability diagram of GL cross-section GR showing the that the triple point considered in the main text is indeed the (0,1)-(1,0) charge transition.

SUPPLEMENTARY NOTE 2: MEASUREMENTS ON A SECOND DOUBLE QUANTUM DOT

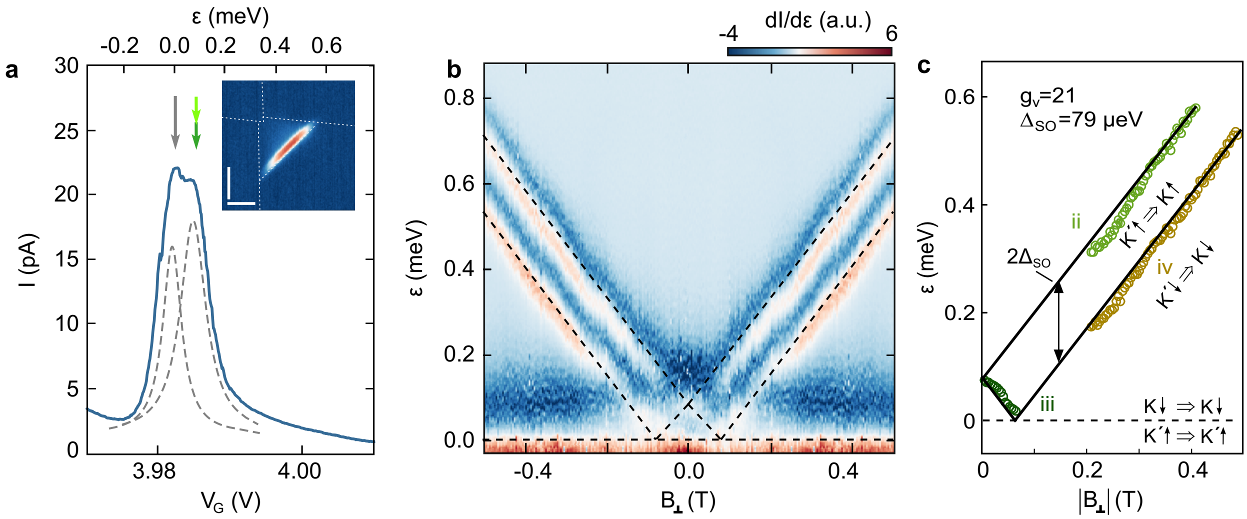

Supplementary Figure 2. **Measurements on a second DQD formed using a different set of gate fingers.** **a** Detuning line cut at zero magnetic field (similar measurement as in Fig. 3d of the main text). Two resonances, one corresponding to transition (i) and one corresponding to transitions (ii) and (iii) are observed. The inset shows the (0,1)-(1,0) triple point, the scale bars correspond to a difference in gate voltage of 20 mV. **b** Magnetotransport measurements showing $dI/d\varepsilon$ as function of detuning energy and out-of-plane B-field. The dashed lines mark the observed interdot transitions. **c** Extracted transition energies as function of $B_\perp$ highlighting the transitions (ii), (iii) and (iv) (see labels).

SUPPLEMENTARY NOTE 3: ESTIMATION OF $\Delta_{\mathrm{KK'}}$

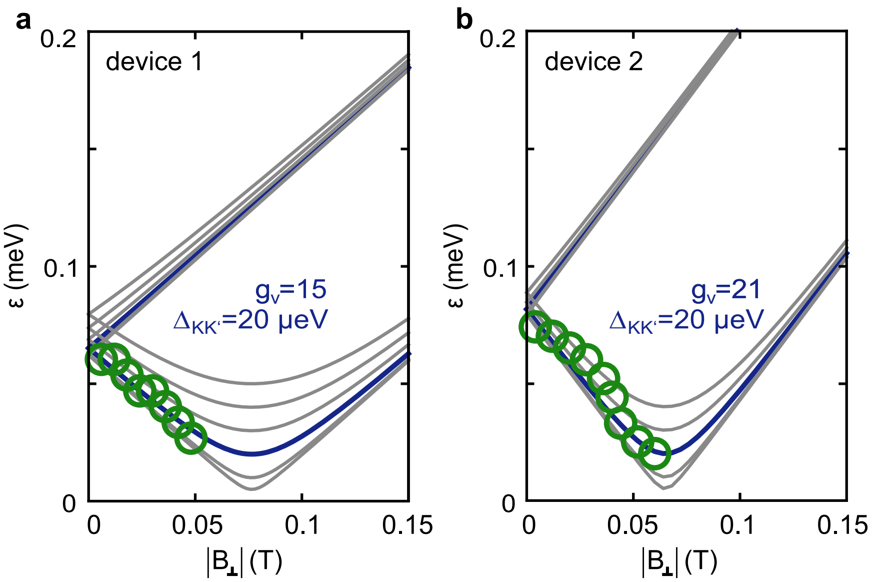

Supplementary Figure 3. **Estimation of the intervalley coupling,** $\Delta_{\mathrm{KK'}}$ for the first (**a**) and second (**b**) double quantum dot measured. The blue lines mark the transition energies, where $\Delta_{\mathrm{KK'}} = 20\ \mu$eV is assumed, which marks an upper bound for $\Delta_{\mathrm{KK'}}$ in both devices.

SUPPLEMENTARY NOTE 4: REFERENCE MEASUREMENTS PERFORMED ON SINGLE QDS

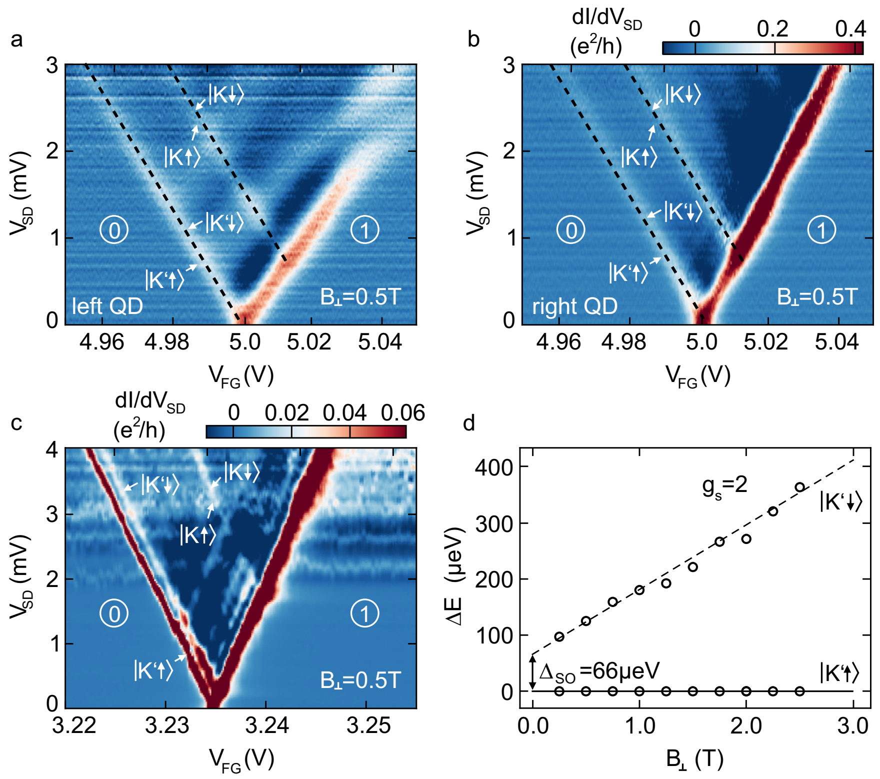

Supplementary Figure 4. **Finite bias spectroscopy measurements.** **a** Finite bias spectroscopy measurement of the single electron spectrum of the left (**a**) and right (**b**) single QD of the device discussed in the main text, recorded at a perpendicular magnetic field of 0.5 T. The valley splitting due to the perpendicular field can be resolved in the two single QDs and very similar for both dots. The tunnel broadening of the Coulomb resonances do not allow to resolve the spin splitting. **c** Finite bias spectroscopy measurement performed on a different device on another chip with a design of more opaque tunneling barriers/less tunnel broadening, recorded at a finite perpendicular magnetic field of $B_\perp$ =0.5 T. Here, the Zeeman spin splitting can be observed. **d** From similar measurement as depicted in c, recorded for various magnetic fields, we extract the Zeeman splitting between $|K'\uparrow\rangle$ and $|K'\downarrow\rangle$. From the data, we extract a spin g-factor of $g_s = 2$ and $\Delta_{\rm SO} = 66 \pm 8\ \mu$eV.
